# SMAD4 activates Wnt signaling pathway to inhibit granulosa cell apoptosis

**DOI:** 10.1038/s41419-020-2578-x

**Published:** 2020-05-15

**Authors:** Xing Du, Qiqi Li, Liu Yang, Lu Liu, Qiuyu Cao, Qifa Li

**Affiliations:** College of Animal Science and Technology, Nanjing Agricultural University, Nanjing, 210095 China

**Keywords:** Apoptosis, Reproductive disorders

## Abstract

The TGF-β and Wnt signaling pathways are interrelated in many cell types and tissues, and control cell functions in coordination. Here, we report that SMAD4, a downstream effector of the TGF-β signaling pathway, induces FZD4, a receptor of the Wnt signaling pathway, establishing a novel route of communication between these two pathways in granulosa cells (GCs). We found that SMAD4 is a strong inducer of *FZD4*, not only initiating *FZD4* transcription but also activating FZD4-dependent Wnt signaling and GC apoptosis. Furthermore, we identified the direct and indirect mechanisms by which SMAD4 promotes expression of *FZD4* in GCs. First, SMAD4 functions as a transcription factor to directly bind to the *FZD4* promoter region to increase its transcriptional activity. Second, SMAD4 promotes production of *SDNOR*, a novel lncRNA that acts as a sponge for miR-29c, providing another mean to block miR-29c from degenerating *FZD4* mRNA. Overall, our findings not only reveal a new channel of crosstalk between the TGF-β and Wnt signaling pathways, SMAD4–FZD4 axis, but also provide new insights into the regulatory network of GC apoptosis and follicular atresia. These RNA molecules, such as miR-29c and lnc-*SDNOR*, represent potential targets for treatment of reproductive diseases and improvement of female fertility.

## Introduction

The canonical transforming growth factor-β (TGF-β) signaling pathway is of great physiological importance. The process of TGF-β signal transduction is quite simple: an extracellular TGF-β signal (ligands, mainly TGF-β1) first interacts with specific membrane receptors (TGFBR1/2) and activates cytoplasmic effector SMAD proteins (SMAD2/3), which further forms a regulation complex with SMAD4 and ultimately translocated into the nucleus, where they drive transcription^1,2^. Among the SMAD proteins, SMAD4 is the only co-mediator. By acting as a central intracellular effector and final downstream component, SMAD4 plays an essential role in TGF-β signal transduction^3,4^. Loss or deficiency of SMAD4 not only inactivates TGF-β signaling, but also impairs TGF-β-mediated transcriptional regulation and biological functions^5,6^. Studies using conditional *Smad4* knockout (*Smad4-*KO) mouse models have revealed that loss of SMAD4 can decrease the sensitivity of tissues or cells to TGF-β1^7,8^.

SMAD4 is not only a mediator and effector of the TGF-β signaling pathway in the nucleus, but is also a unique multifunctional modulator that regulates both transient and persistent cellular processes including cell proliferation^9^, differentiation^10^, autoimmunity^11^, pluripotency and plasticity^12^ and cell growth^13^, apoptosis^14^, autophagy^15^, invasion^16^, and metastasis^17^. SMAD4 dysregulation is associated with embryonic developmental disorders^18^, deficiencies in skeletal muscle differentiation and regeneration^19^, loss of stem cell pluripotency^20^, disorder and delay of nervous system development^21^, female infertility^22^, and other pathologies. Furthermore, deficiency or inactivation of SMAD4 diminishes innate immune responses to viral infection^23^, promotes inflammation^24^, and can even drive cancers^25^. Accordingly, SMAD4 has been identified in quantitative studies as a biomarker of multiple cancers and probed extensively as a potential pharmaceutical target^26^. In ovary, SMAD4 and SMAD4-dependent TGF-β signaling pathway have shown to be associated with follicular development, especially follicular atresia, the ultimate fate of most follicular development^27,28^. Indeed, recent reports demonstrated that SMAD4 contributes to follicular atresia through inhibiting granulosa cell (GC) apoptosis, which is the main inducement of follicular atresia^29^. However, the mechanism of SMAD4 regulating GC apoptosis is not completely understood.

As a multifunctional intracellular signal transducer, SMAD4 has multiple functions, some but not all of which are dependent on its DNA-binding capacity, in various contexts it can behave as a transcriptional activator, co-regulator, or nuclear localization factor^10,23^. SMAD4 is perhaps best known as a transcription factor, and in regulation of some of its target genes, it interacts with other cooperative factors such as co-activators and co-repressors^30,31^. SMAD4-binding elements (SBEs) in the promoter region are necessary for SMAD4 to recognize target genes and regulate transcription initiation^32^. The best-characterized SBE motifs are GTCTG, CAGAC, and the newly identified 5 bp GC-rich sequence GGC(GC)/(CG)^33,34^. In recent years, many direct targets of SMAD4, including both coding and noncoding genes (i.e., miRNAs and long noncoding RNAs), have been identified using chromatin immunoprecipitation (ChIP), ChIP-seq, and genome-wide mapping technology^35,36^. In our previous study, we characterized the transcriptome of porcine GCs in which SMAD4 was silenced, and identified FZD4, a receptor of the Wnt signaling pathway and regulator of GC functions^37^, as a new candidate target of SMAD4^38^. In this study, we sought to confirm the regulatory effect of SMAD4 on FZD4 in GCs and to elucidate the molecular mechanism underlying this regulation, and their roles in regulating GC apoptosis.

## Materials and methods

### Cell culture and treatment

Porcine GCs were isolated and cultured as previously described^39^. Briefly, GCs were collected from 3-5 mm of healthy follicles with 22-gauge needles and seeded into T25 flasks with Dulbecco’s Minimum Essential medium/nutrient F-12 (DMEM/F-12, Gibco) containing 10% fetal bovine serum (FBS) (Gibco), 100 units/mL penicillin, and 100 mg/mL streptomycin (Gibco). HEK 293T cells were cultured in DMEM with 10% FBS at 37 °C in a 5% CO_2_ incubator. Lipofectamine^®^ 3000 transfection reagent (#L3000015, Life Technologies, Carlsbad, CA 92008 USA) was used to transfect oligonucleotides or plasmids at a final concentration of 20 μM. The oligonucleotides used in this study are listed in Supplementary Table S1. Animal experiments were approved by the Animal Ethics Committee at Nanjing Agricultural University, Nanjing, P. R. China (SYXK 2017-0027) and performed in accordance with the Regulations for the Administration of Affairs Concerning Experimental Animals (No. 2 of the State Science and Technology Commission, 11/14/1988).

### Bioinformatic analysis

The candidate SMAD4-binding sites in the promoter of *FZD4* and SMAD4-dependent noncoding RNA (*SDNOR*) were predicted by JASPAR (jaspar.genereg.net/), a software for prediction the binding motifs of transcription factors. Potential miRNAs that target *FZD4* were predicted by four different algorithms, miRWalk 3.0 database (http://zmf.umm.uni-heidelberg.de/apps/zmf/mirwalk3/), miRDB (http://www.mirdb.org/miRDB/), TargetScan (http://www.targetscan.org/), and miRTarBase (http://amp.pharm.mssm.edu/Harmonizome/resource/MiRTarBase). RNAhybrid (http://bibiserv.techfak.uni-bielefeld.de/rnahybrid/) was performed to predict miR-29c binding sites in pig *FZD*4 3′-UTR and *SDNOR*. miRBase (http://www.mirbase.org/) was used to obtain pre- and mature miRNAs sequences. The coding potential of *SDNOR* was predicted by two software, Coding Potential Assessment Tool and Coding Potential Calculator.

### Plasmids construction and dual-luciferase reporter assays

To generate luciferase reporters, the fragments of *FZD4* and *SDNOR* promoters were amplified from porcine genomic DNA and cloned into pGL3-basic vector. The fragments of *SDNOR* and the 3′-UTR of *FZD4* that contained putative miR-29c binding sites were cloned into pmirGLO vector. Mutant vectors were generated using the TaKaRa MutanBEST Kit (#R401, TaKaRa, Beijing, China). All the recombinant plasmids were verified by Sanger sequencing. Primers used for plasmids construction are listed in Supplementary Table S2.

After transfection for 24–48 h, porcine GCs were harvested and the lysates were collected for dual-luciferase analysis by using the Dual-Luciferase Reporter Assay System (#E1910, Promega, Madison, USA) following the kit’s manual. The GLOMAX detection system (Promega) was conducted to measure the firefly and renilla luciferase activities in cell lysates.

### Rapid amplification of cDNA end (RACE)

The full-length sequence of the *SDNOR* transcript and the 5′-flanking sequence of *FZD4* were obtained by using the SMARTer^®^ RACE 5′/3′ Kit (#634858, Clontech Laboratories, Inc, CA94043, USA). Briefly, total RNA from porcine GCs was reverse-transcribed into first-strand cDNA using SMARTScribe reverse transcriptase. cDNAs were then amplified, ligated to adapters, and cloned into pUC19 vector. The full-length sequence of *SDNOR* and the 5′-flanking sequence of *FZD4* were confirmed by Sanger sequencing. The primers used in this process are listed in Supplementary Table S3.

### Quantitative real-time PCR assay

In brief, total RNA was isolated from cells using the High-Purity RNeasy Mini Kit (#74104, Qiagen, Beijing, China) and reverse-transcripted into cDNA by using HiScript^®^ II Q RT SuperMix for qPCR (#R223-01, Vazyme Biotech Co., Ltd, Nanjing, China) according to the manufacturer’s instruction. Quantitative real-time PCR (qRT-PCR) analysis was performed using the StepOnePlus System (Applied Biosystems) with AceQ qPCR SYBR Green Master Mix (#Q111-02, Vazyme Biotech Co., Ltd, Nanjing, China). Fold changes of interested genes were computed using the 2^−ΔΔCt^ method. qRT-PCR was conducted in triplicate, and the results are presented as mean ± S.E.M. after normalization to *GAPDH* and *U6* for coding and noncoding genes, respectively. Primers used for real-time PCR are listed in Supplementary Table S4.

### Subcellular localization

Nuclear and cytoplasmic were extracted from porcine GCs using the method as previously described4^40^. Briefly, porcine GCs were lysed in cold lysis buffer and placed on ice for 10 min. Then, cells were centrifuged at 12,000 × *g* for 3 min at 4 °C. The supernatant (cytoplasmic extract) was immediately frozen (−80 °C) for subsequent analysis. The nuclear pellet was resuspended with cold DEPC water containing 1 mM RNase inhibitor and placed on ice for 5 min, and then centrifuged at 10,000 × *g* for 5 min. The supernatant (nuclear extract) was removed and the remainder was frozen (−80 °C) for subsequent analysis.

### Western blotting

For western blotting analysis, protein lysates from whole cells were prepared using RIPA buffer with protease inhibitors and phosphatase inhibitors. After incubation on ice for 30 min, the supernatant was collected by 12,000 × *g* centrifugation for 15 min at 4 °C. The BCA Protein Assay Kit (#P0012, Beyotime, Jiangsu, China) was used to detect the concentration of total protein and western blotting was conducted as described previously^41^. Primary antibodies were anti-FZD4 (Sangon Biotech, #D121422, 1:1000), anti-β-catenin (Sangon Biotech, #D260137, 1:1000), anti-caspase-3/cleaved caspase-3 (Proteintech, #19677-1-AP, 1:1000), and anti-GAPDH (ORIGENE, #TA802519, 1:5000). HRP-conjugated secondary antibodies were diluted in 0.25% BSA/TBST. The original high-resolution western blotting images were obtained by a high-sensitivity chemiluminescence imaging system (Bio-rad, #Chemi DOC touch) and the densitometry of each blotting image was analyzed by Quantity One software with Gauss Model Trace method. House-keeping protein GAPDH was used as an internal control.

### Flow cytometry

Porcine GC apoptosis was detected using the Annexin V-FITC/PI Apoptosis Detection Kit (#A211-01, Vazyme Biotech Co., Ltd, Nanjing, China), and flow cytometry was performed as previously described^40^. Cells were counted by flow cytometry (Becton Dickinson), and the rate of apoptosis was analyzed using the FlowJo software (TreeStar). Specifically, the rate of apoptosis was calculated based on the percentage of cells in the Q2 and Q3 quadrants, representing early- and late-stage apoptotic cells, respectively.

### Chromatin immunoprecipitation

ChIP assays were performed as previously described^39^. The SMAD4–DNA complex was pulled down with rabbit anti-SMAD4 (Santa Cruz Biotechnology, #sc-1909-R) antibody. After decrosslinking, enrichment of interested DNA fragments was analyzed by semiquantitative PCR and qPCR. Antibody against IgG (Santa Cruz, #sc-2358) was used as the internal control, and unprocessed chromatin served as the input control. PCR primers used in these experiments are listed in Supplementary Table S5. ChIP-qPCR signals were calculated as fold enrichment relative to input control signals; experiments were performed in technical triplicates. Specific antibody ChIP signals were normalized against IgG control ChIP signals from the same samples.

### RNA pull-down

*SDNOR*-WT (miR-29c MRE1, MRE2), *SDNOR*-MUT (miR-29c MRE1, MRE2), and *NORFA*-WT (miR-126 MRE) were transcribed from vector pSPT19-*SDNOR*-WT, pSPT19-*SDNOR*-MUT, and pSPT19-*NORFA*-WT in vitro, respectively. Five single-stranded RNA transcripts were biotinylated modified by Biotin-RNA Labeling Mix (#No. 11685597910, Roche) and T7 RNA polymerase (#EP0111, Thermo Fisher Scientific), which were then collected and purified with an RNeasy Mini Kit (#74104, Qiagen). The purified biotinylated transcripts (4 μg) were incubated with 15 μg porcine GC total RNA for 4 h at room temperature. Streptavidin magnetic beads (#LSKMAGT02, Merck Millipore) were used to isolate the biotin-RNA/interested-RNA complex according to the manufacturer’s protocol. After isolation, the levels of target miRNAs presenting in the pull-down material were detected by qRT-PCR analysis.

### Statistical analysis

All statistical evaluations were performed using GraphPad Prism v5.0 software (GraphPad software) and the Statistical Program for Social Sciences software v20.0 (SPSS). The Student’s *t* test (two groups) and one-way analysis of variance (ANOVA) were used for single comparison and multiple group comparisons, respectively. Multiple comparisons between the groups after ANOVA test were confirmed by using S–N–K method. *P < 0.05 was considered statistically significant.

## Results

### SMAD4 is a strong inducer of *FZD4* in porcine GCs

**Fig. 1:**
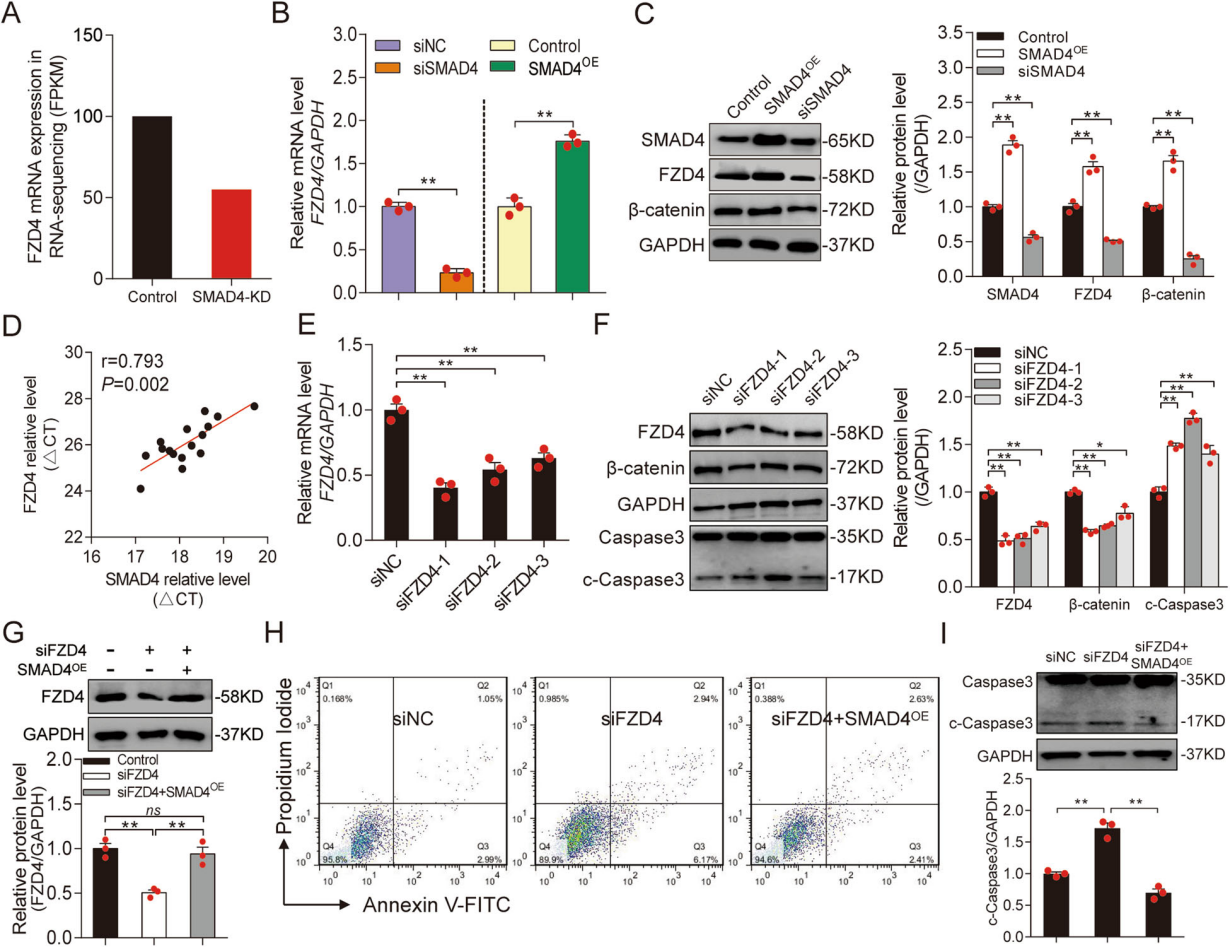
*FZD4* is significantly upregulated by SMAD4 in porcine GCs. **a** *FZD4* mRNA expression level (fragments per kb of exon per million fragments mapped; FPKM) in porcine GCs under treatment with siRNA (control) or siSMAD4 (SMAD4-KD) according to RNA-seq. **b** *FZD4* mRNA expression in porcine GCs under treatment with SMAD4-siRNA (siSMAD4) or SMAD4-overexpressing vector (SMAD4^OE^) was assessed by qRT-PCR. **c** FZD4 and β-catenin protein levels in porcine GCs after SMAD4 overexpressed or knockdown were measured by western blot (left), quantification was shown in right panel. GAPDH was used as a loading control. **d** The correlation between *SMAD4* and *FZD4* mRNA level was detected in 15 porcine ovarian follicles. The ΔCt values were subjected to Pearson correlation analysis. **e** qRT-PCR validation of *FZD4* mRNA levels in porcine GCs after specific FZD4 knockdown with two small-interfering RNAs against siFZD4-1 and siFZD4-2. **f** Representative western blot analysis (left) and quantification (right) of FZD4, β-catenin and cleaved Caspase-3 (c-Caspase-3) protein levels in FZD4-silenced porcine GCs. GAPDH was used as a loading control. **g** FZD4 protein levels in porcine GCs co-transfected with siFZD4 and SMAD4^OE^ were analyzed by western blot. Images (top) and quantification (bottom). **h** Representative FACS analysis of the apoptosis rate of porcine GCs after FZD4 knockdown or with SMAD4 ectopic expression. **i** Representative western blot images (top) and analysis (bottom) for c-Caspase-3 expression in porcine GCs treated with siFZD4 or siFZD4 with SMAD4 overexpression. Con. indicates control. Throughout, results are shown as mean ± S.E.M. (*n* = 3, each). **p < 0.01, ns indicates no significance versus scrambled or control.

In a previous study, we used RNA-Seq to identify 1025 mRNAs that were differentially expressed (greater than twofold) in SMAD4 knockdown (SMAD4-KD) porcine GCs^38^. Among them was *FZD4*, which encodes an important receptor in the Wnt signaling pathway (Fig. 1a). qRT-PCR demonstrated that the *FZD4* mRNA level was dramatically reduced in SMAD4-KD GCs, but significantly induced in cells overexpressing SMAD4 (SMAD4-OE) (Fig. 1b). Consistent with this, the level of FZD4 was markedly decreased by SMAD4-KD and significantly increased by SMAD4-OE (Fig. 1c), indicating that SMAD4 is a strong inducer of FZD4 in porcine GCs. Furthermore, β-catenin, the key downstream molecule of FZD4 and mediator of the Wnt signaling pathway, was also positively regulated by SMAD4 (Fig. 1c), suggesting that SMAD4 controls the Wnt signaling pathway in porcine GCs. In addition, we noticed that expression of SMAD4 was positively correlated with FZD4 expression (Pearson correlation coefficient *r* = 0.793, *p* = 0.002) in follicles of porcine ovary in vivo (Fig. 1d). Together, these data suggest that SMAD4 induces FZD4 and the FZD4-dependent Wnt signaling pathway in porcine GCs.

Interestingly, we also noticed that FZD4 is related to porcine follicular atresia in vivo (Fig. S1). To further explore the biological implications of FZD4 in porcine GCs, we knocked down endogenous FZD4, resulting in attenuation of the Wnt signaling pathway in these cells (Fig. 1e–g). Notably, FZD4 depletion dramatically induced cleaved Caspase-3 (c-Caspase-3), a marker of apoptosis (Fig. 1f). Furthermore, FACS revealed that FZD4 depletion significantly induced GC apoptosis (Fig. 1h), indicating that FZD4, like its inducer SMAD4, is an antiapoptotic factor in porcine GCs^41^.

We next investigated whether SMAD4 regulates FZD4-mediated GC function, and found that ectopic expression of SMAD4 rescued FZD4 protein level and GC apoptosis rate due to FZD4 depletion caused by siFZD4 (Fig. 1g, h). Consistent with this, the presence of SMAD4 inhibited induction of c-Caspase-3 following FZD4 depletion (Fig. 1i). Together, these findings strongly suggest that SMAD4 initiates FZD4 expression and activates the Wnt signaling pathway, which then suppresses apoptosis, in porcine GCs.

### SMAD4 induces *FZD4* by directly binding its promoter

**Fig. 2:**
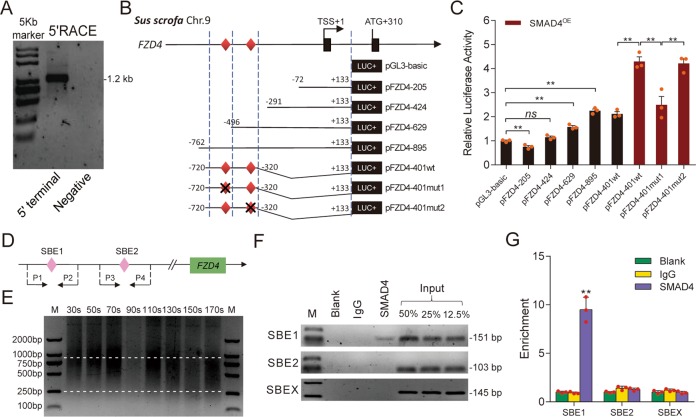
SMAD4 acts as a transcription factor and induces *FZD4* transcription. **a** Representative 5′ RACE analysis for detection the 5′ terminal of *FZD4* transcript. **b** Schematic showing that different loci of *FZD4* promoter were constructed into pGL3 vector. Potential SBE sites were indicated by red diamonds and transcription start sit (TSS) was counted as +1. The mut indicates SBE mutation. **c** The activities of recombinant luciferase reporters in porcine GCs with or without SMAD4 overexpression were measured. **d** Schematic diagram depicting the primers for ChIP assays. P1-P4: primers used for ChIP and ChIP-qPCR assays. **e** Identification of the optimal ultrasonic time for ChIP assay. Different ultrasonic times (30–170 s) were chosen to acquire the appropriate chromatin size (250–1000 bp). **f** Representative ChIP analysis for *FZD4* promoter following immunoprecipitation with SMAD4-specific antibody or a nonspecific IgG antibody. Blank indicates no antibody addition. Input titrations are shown for each chromatin preparation (50, 25, and 12.5%). **g** ChIP-qPCR was performed to detect SMAD4 endogenously associated with SBEs in *FZD4* promoter. Data in **c** and **g** are represented by mean ± S.E.M. (*n* = 3, each). **p < 0.01 and ns indicates no significance versus scrambled, control, or input.

It has been reported that SMAD4 regulates transcription of coding and noncoding genes by acting as a transcription factor^39,42^, we hypothesized that SMAD4 initiates *FZD4* transcription via the same mechanism. To test this idea, we determined the transcription start site (TSS) of the porcine *FZD4* gene through RACEs, and detected only one clear PCR band with a length of ~1.6 kb (Fig. 2a). Clone sequencing and BLAST revealed that porcine *FZD4* has one TSS, located 310 nt upstream of the start codon (ATG) (Figs. 2b and S2). Luciferase assays using deletion constructs (Fig. 2b) revealed that the DNA region from 762 to 496 nt (TSS was defined as +1) is the core promoter of porcine *FZD4* (Fig. 2c). In addition, SMAD4-OE significantly increased core promoter activity (Fig. 2b, c), suggesting that SMAD4 controls *FZD4* transcription via the core promoter.

Interestingly, we identified several binding sites for transcription factors, including EGR1, E2F4, FOXD2, and SMAD4. Two SBEs, designated SBE1 (-504/-501) and SBE2 (-325/-323), are present in the core promoter region of porcine *FZD4* (Figs. 2b and S2). Overexpression of SMAD4 significantly increased the luciferase activity of *FZD4* promoter constructs containing SBE1, SBE2, or mutated SBE2, but had no effect on the activity of a SBE1-mutated promoter (Fig. 2c). ChIP and ChIP-qPCR assay confirmed that SMAD4 could directly bind to SBE1, but not to SBE2 (Fig. 2d–g). Altogether, the observations above provide compelling evidence that SMAD4 induces *FZD4* transcription in porcine GCs by directly interacting with the SBE1 motif in its promoter.

### miR-29c acts as an apoptotic factor by suppressing FZD4 expression in porcine GCs

**Fig. 3:**
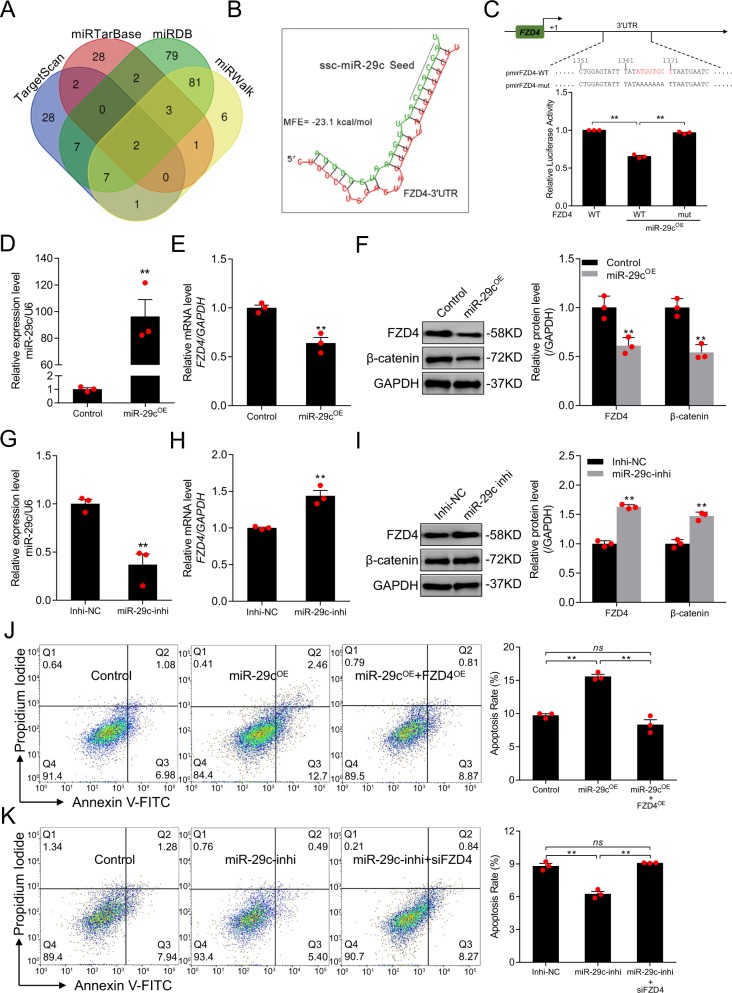
miR-29c attenuates FZD4 expression and induced porcine GC apoptosis. **a** Potential miRNAs targeting FZD4 were predicted through four different algorithms. **b** Diagram showing the sequence of putative miR-29c binding site in *FZD4* 3′UTR and MFE (minimum free energy). Black line indicated miR-29c seed sequence. **c** Recombinant reporter vectors containing MRE wild-type (WT), mutated *FZD4* 3′UTR (top), and their luciferase activities in HEK293T cells with or without miR-29c were calculated (bottom). miR-29c levels (**d**) and *FZD4* mRNA levels (**e**) in porcine GCs treated with miR-29c mimcis (miR-29c^OE^) were assessed by qRT-PCR. **f** Western blot analysis (left) and quantification (right) for FZD4 and β-catenin protein levels in porcine GCs with miR-29c overexpression. GAPDH serves as control. miR-29c levels (**g**) and *FZD4* mRNA levels (**h**) in porcine GCs treated with miR-29c inhibitor (miR-29-inhi) were assessed by qRT-PCR. **i** Western blot analysis (left) and quantification (right) for FZD4 and β-catenin protein levels in pGCs transfection with miR-29c knockdown. GAPDH serves as control. FACS analysis (left) and quantification (right) for the apoptosis rate of porcine GCs after miR-29c mimics treatment or with FZD4 overexpression (FZD4^OE^) (**j**), and miR-29c inhibitor addition or with FZD4 knockdown (**k**). Data in **c**–**k** are represented as mean ± S.E.M. (*n* = 3, each). **p < 0.01, ns indicates no significance versus control or scrambled.

The SMAD4-dependent miRNA–mRNA interaction network suggested that two miRNAs, miR-29c and miR-10b, may target FZD4^39^. We identified candidate miRNAs targeting *FZD4* using four algorithms (miRWalk, miRDB, miRTarBase, and TargetScan), and identified miR-29c as one of two common miRNAs (the other was let-7g) (Fig. 3a). Therefore, we selected miR-29c for further study. Using RNAhybrid, we identified a putative miRNA response element (MRE) for miR-29c in the 3′-UTR of porcine *FZD4* (Fig. 3b), and a minimum free energy (MFE) approach confirmed that miR-29c had a high binding capacity for the *FZD4* 3′-UTR. Moreover, the mature and seed sequence of miR-29c and the MRE within the *FZD4* 3′-UTR are highly evolutionarily conserved in vertebrates (Fig. S3). In a luciferase activity assay, miR-29c dramatically decreased the luciferase activity of pmirFZD4-WT (Fig. 3c). Conversely, the luciferase activity of pmirFZD4-mut, which harbors a MRE mutation, did not change under miR-29c treatment (Fig. 3c). These data indicate that miR-29c directly targets porcine *FZD4* via its 3′-UTR region.

In vivo, miR-29c and *FZD4* mRNA levels were negatively correlated (*r* = −0.6151, *p* = 0.033) in follicles of porcine ovary (Fig. S4). To determine the effects of miR-29c on endogenous FZD4 expression in porcine GCs, we performed loss- and gain-of-function experiments (Fig. 3d, g). Ectopic expression of miR-29c dramatically decreased the levels of *FZD4* mRNA (Fig. 3e) and FZD4 protein (Fig. 3f), as well as the level of β-catenin (Fig. 3f), in GCs. By contrast, the levels of *FZD4* mRNA (Fig. 3h), FZD4 protein, and β-catenin protein (Fig. 3i) were significantly increased by miR-29c depletion. These data suggest that FZD4 is a functional target of miR-29c in GCs, and that miR-29c inhibits both FZD4 and the FZD4-dependent Wnt signaling pathway.

We next investigated the role of miR-29c in porcine GCs. FACS revealed that ectopic expression of miR-29c significantly induced GC apoptosis (Fig. 3j), whereas inhibition of miR-29c significantly decreased it (Fig. 3k), indicating that miR-29c acts as a pro-apoptotic factor in porcine GCs. Furthermore, miR-29c-induced GC apoptosis was prevented by FZD4-OE (Fig. 3j). Depletion of FZD4 restored GC apoptosis in cells treated with miR-29c inhibitor (Fig. 3k). In addition, miR-29c significantly elevated the level c-Caspase-3, and this effect was reversed by ectopic expression of FZD4 (Fig. S5). Together, these results prove that miR-29c directly targets FZD4 and inhibits its expression, leading to apoptosis.

### *SDNOR*, a novel SMAD4-dependent lncRNA, is involved in follicular atresia and GC apoptosis

**Fig. 4:**
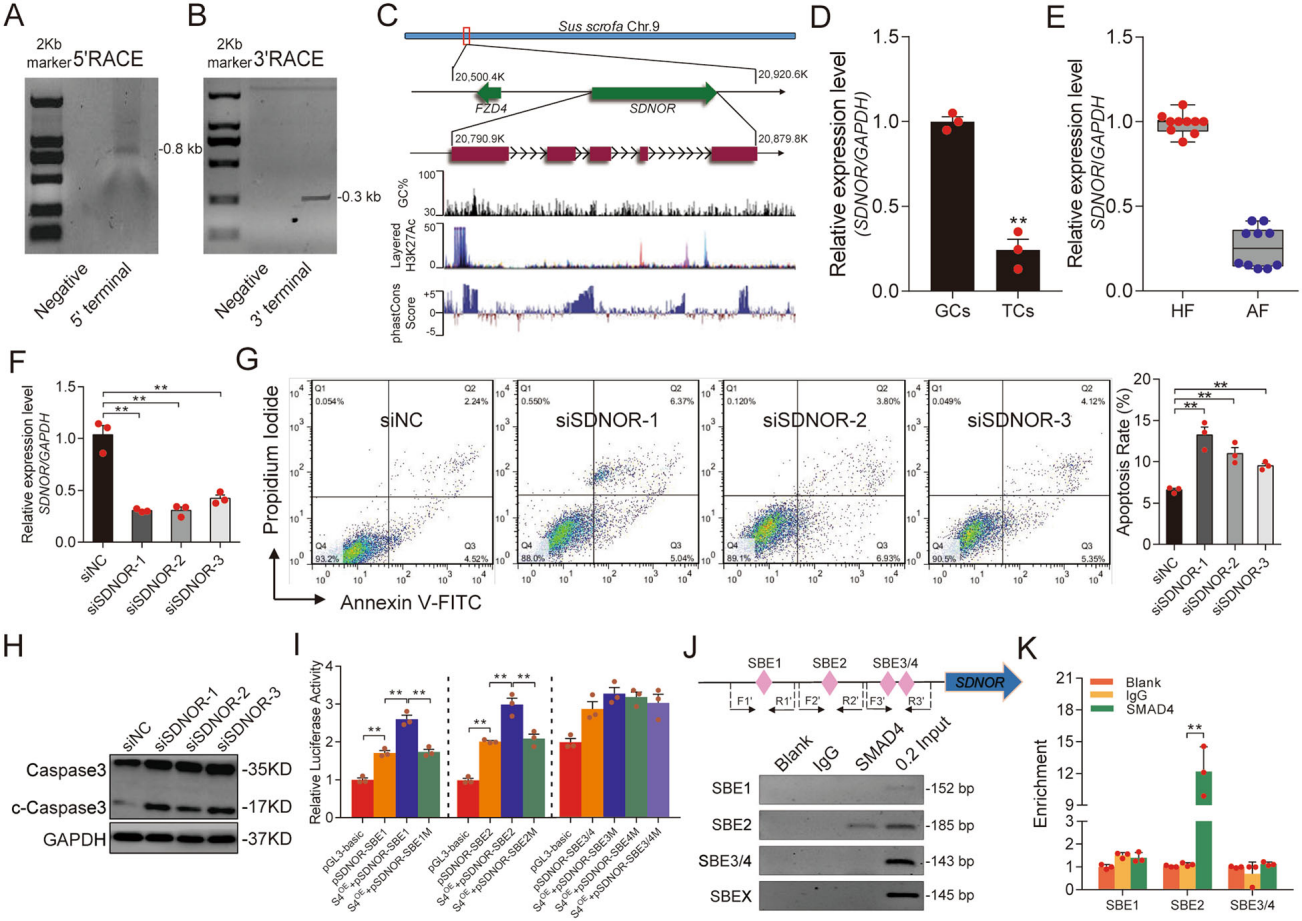
*SDNOR*, a novel antiapoptotic lncRNA, is regulated by SMAD4. Detection of 5′ and 3′ terminals of the novel lncRNA by using 5′RACE (**a**) and 3′RACE (**b**). **c** Schematic annotation of *SDNOR* with associated UCSC Genome Browser tracks depicting genomic locus, GC percent, H3K27Ac modification as well as mammalian conservation. **d** qRT-PCR validation of *SDNOR* expression levels in pig ovarian GCs and TCs (Theca cells). **e** Detection of *SDNOR* expression in healthy follicles (HF) and atresic follicles (AF), assessed by qRT-PCR (*n* = 10). **f** *SDNOR* expression levels in porcine GCs after *SDNOR* knockdown, determined by qRT-PCR. **g** FACS analysis (left) and quantification (right) of the apoptosis rate of porcine GCs after *SDNOR* knockdown. **h** Representative western blot analysis (left) and quantification (right) of c-Caspase-3 protein levels in GCs after *SDNOR* knockdown. **i** Luciferase activities of reporter vectors containing pig *SDNOR* promoter in porcine GCs with or without SMAD4 overexpression (S4^OE^) were assessed. **j** Diagram depicting *SDNOR* promoter (top) and ChIP assay (down), SBE shown as pink diamonds; F1′/R1′, F2′/R2′, and F3′/R3′: primers used for ChIP and ChIP-qPCR assay. **k** ChIP-qPCR were performed to detect the different SBEs in the promoter of *SDNOR* following immunoprecipitation with a SMAD4 antibody. Throughout, all experiments were performed in triplicate. Data in **d**–**k** are shown as mean ± S.E.M. (*n* = 3, each). **p < 0.01 versus control or scrambled.

To further investigate the mechanism underlying SMAD4 inhibition of miR-29c, we deduced a SMAD4-dependent lncRNA–miR-29c interaction network from our previous RNA-seq data^38^. Notably, a novel lncRNA not only interacts with miR-29c, but also maps near *FZD4* on porcine chromosome 9 was identified. We performed a RACE assay to obtain the full-length sequence of this lncRNA, and found that it is 739 nt in length (Figs. 4a, b and S6a). Based on bioinformatic prediction, this lncRNA has no protein-coding potential (Fig. S6b, c); accordingly, we named it *SDNOR*. Porcine *SDNOR* is located ~69.5 kb upstream of *FZD4* (chromosome 9) and consists of five exons and four introns that exhibit relatively low evolutionary conservation among mammals (Fig. 4c). In addition, we identified an enrichment in H3K27 acetylation at the TSS of porcine *SDNOR*, suggesting that it has the features of an RNA polymerase II transcription unit (Fig. 4c).

Tissue expression profiling of the reproductive system revealed that *SDNOR* is abundantly expressed in porcine ovary (Fig. S6d). In ovarian follicles, *SDNOR* was mainly present in GCs (Fig. 4d). Notably, we also observed that *SDNOR* was dramatically downregulated during follicular atresia (Fig. 4e), suggesting that it is somehow involved in this process. To further assess the role of *SDNOR* in regulating follicular atresia, we knocked down endogenous *SDNOR* in porcine GCs cultured in vitro (Fig. 4f). Silencing of *SDNOR* significantly increased GC apoptosis (Fig. 4g) and the level of c-Caspase-3 (Fig. 4h). These results suggest that lncRNA *SDNOR* is involved in GC apoptosis and follicular atresia in porcine ovaries.

RNA-seq and qRT-PCR confirmed that *SDNOR* was downregulated in SMAD4-silenced porcine GCs (Fig. S7a, b), indicating that SMAD4 inhibits *SDNOR* transcription in these cells. To investigate the mechanism by which SMAD4 regulates *SDNOR* transcription, we first cloned the 5′-regulatory region containing the putative promoter of porcine *SDNOR*. Four SBE motifs were identified at 984/988 nt (SBE1), 776/779 nt (SBE2), 364/467 nt (SBE3), and 341/344 nt (SBE4) (Fig. S7c). Luciferase assays proved that SMAD4 ectopic expression could significantly increase the activity of the *SDNOR* promoter, and that the SBE1 and SBE2 motifs are required to maintain this situation (Figs. 4i and S7d). In addition, ChIP assays confirmed that SMAD4 directly binds to SBE2 within the *SDNOR* promoter by functioning as a transcription factor (Fig. 4j, k). Together, these observations demonstrate that SMAD4 acts as a transcriptional regulator of *SDNOR* in porcine GCs.

### SMAD4 promotes expression of FZD4 by inducing *SDNOR*

**Fig. 5:**
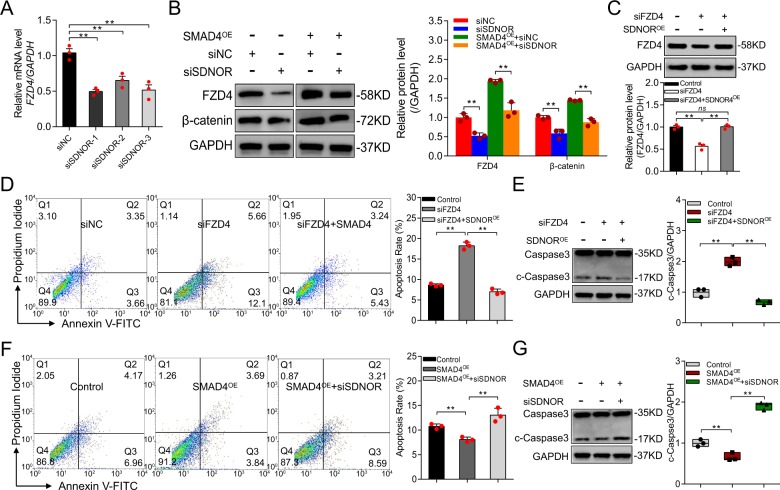
SMAD4 regulates FZD4 expression via *SDNOR*. **a** *FZD4* mRNA levels in porcine GCs after *SDNOR* knockdown, assessed by qRT-PCR. **b** Western blot analysis (left) and quantification (right) of FZD4, β-catenin protein levels in *SDNOR*-KD porcine GCs or in SMAD4^OE^ pGCs after *SDNOR* depletion, *GAPDH* used as control. **c** Western blotting analysis (top) and quantification (bottom) of FZD4 protein level in porcine GCs co-transfected siFZD4 with *SDNOR*^OE^. **d** FACS analysis (left) and quantification (right) of the apoptosis rate of porcine GCs after FZD4 knockdown or with *SDNOR* overexpression. **e** Western blot analysis (left) and quantification (right) of c-Caspase-3 protein levels in porcine GCs after FZD4 knockdown or with *SDNOR* ectopic expression, GAPDH used as control. **f** FACS analysis (left) and quantification (right) of the apoptosis rate of porcine GCs after SMAD4 ectopic expression or with *SDNOR* knockdown. **g** Western blot analysis (left) and quantification (right) of c-Caspase-3 protein levels in porcine GCs after SMAD4 overexpression or with *SDNOR* knockdown, *GAPDH* used as control. Throughout, all data are shown as mean ± S.E.M. (*n* = 3, each). **p < 0.01 versus control or scrambled.

lncRNAs often exert their biological functions by controlling nearby genes^43–45^. We noticed that *SDNOR* depletion dramatically decreased the level of *FZD4* mRNA, which is encoded by a gene near *SDNOR*, in porcine GCs (Fig. 5a). Similarly, the FZD4 protein level was significantly downregulated in *SDNOR*-KD GCs (Fig. 5b), indicating that *SDNOR* induces FZD4 expression in porcine GCs. To determine whether *SDNOR* regulates the FZD4-dependent Wnt signaling pathway, we monitored the β-catenin protein level in *SDNOR*-KD GCs. Knockdown of *SDNOR* dramatically decreased the β-catenin protein level (Fig. 5b). In addition, *SDNOR*-OE inhibited FZD4-KD-induced GC apoptosis (Fig. 5c) and production of c-Caspase-3 (Fig. 5d), whereas the opposite effect was observed in GCs co-treated with *SDNOR*-KD and FZD4-OE (Fig. S8a–c). These data demonstrate that *SDNOR* induces FZD4 and the FZD4-dependent Wnt signaling pathway, and controls FZD4-mediated apoptosis, in porcine GCs.

We next investigated whether *SDNOR* is involved in the maintenance of FZD4 expression in porcine GCs. Silencing of *SDNOR* suppressed the SMAD4-induced increases in FZD4 and β-catenin protein levels (Fig. 5b), indicating that *SDNOR* mediates SMAD4 activation of FZD4 and the FZD4-dependent Wnt signaling pathway in porcine GCs. Furthermore, *SDNOR*-KD inhibited the SMAD4-induced downregulation of GC apoptosis (Fig. 5e). In addition, c-Caspase-3 level was increased by *SDNOR*-KD in SMAD4-OE GCs (Fig. 5f), and the opposite effect was observed in GCs co-treated with SMAD4-KD and *SDNOR*-OE (Fig. S8d–f). Together, these findings reveal that *SDNOR* contributes to the maintenance of FZD4 expression in porcine GCs.

### *SDNOR* induces FZD4 by sponging miR-29c

**Fig. 6:**
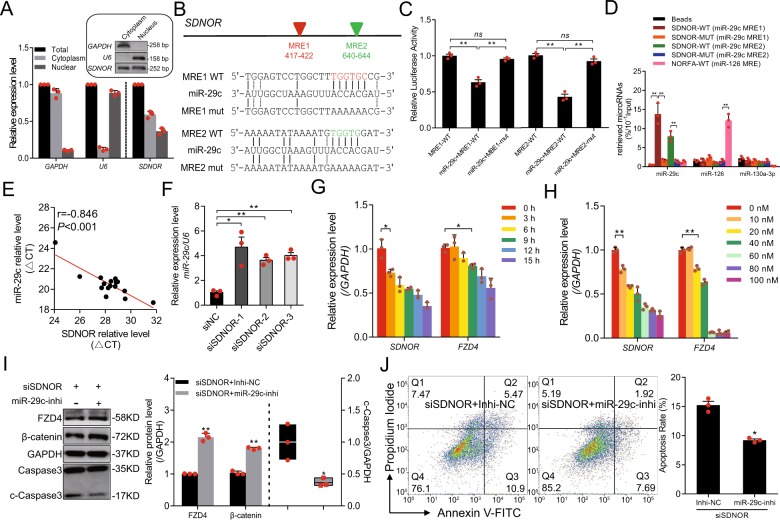
*SDNOR* maintains the expression of FZD4 by physically interacting with miR-29c. **a** Subcellular localization of *SDNOR* in porcine GCs, detected by qRT-PCR. The gel images in the box showing the semi-qPCR results. *GAPDH* and *U6* served as markers for cytoplasm and nucleus, respectively. **b** Schematic diagram represents *SDNOR* transcript (top) and recombinant reporter vectors with wild-type or mutant miR-29c response elements (MRE) (bottom). Predicted miR-29c MREs shown with colored triangles. **c** Luciferase activities of reporter vectors in porcine GCs with or without miR-29c. **d** RNA pull-down analysis for miR-29c following pull-down with biotin-labeled SDNOR, further assessed by qRT-PCR. miR-126 and miR-130a-3p here were used as negative controls. **e** *SDNOR* and *miR-29c* expression levels in 16 porcine ovarian follicles were detected and subjected to Pearson correlation analysis. **f** miR-29c expression levels in porcine GCs after *SDNOR* knockdown, determined by qRT-PCR. *SDNOR* and *FZD4* mRNA levels in porcine GCs transfected with miR-29c for different time (**g**, 0–15 h) or at different concentration (**h**, 0–100 nM), assessed by qRT-PCR. **i** Western blot analysis (left) and quantification (right) of FZD4, β-catenin and c-Caspase-3 protein levels in porcine GCs after *SDNOR* and miR-29c knockdown. *GAPDH* used as a loading control. **j** FACS analysis (left) and quantification (right) of the apoptosis rate of porcine GCs after *SDNOR* and miR-29c knockdown. Data in **a**, **c**, **d**, and **f**–**i** are shown as mean ± S.E.M. (*n* = 3, each). *p < 0.05, **p < 0.01 and ns indicates no significance versus to control or scrambled.

The mechanism of action of a lncRNA usually depends on its subcellular localization^46^. To elucidate the mechanism by which *SDNOR* upregulates FZD4, we first determined the subcellular localization of *SDNOR* in porcine GCs. *SDNOR* was enriched in both the nucleus and cytoplasm (Fig. 6a). Function as molecular sponges, is common mechanism of both nuclear and cytoplasmic lncRNAs;^46^ interestingly, two putative binding sites (TGGTGC, 417–422 nt; TGGTG, 640–422 nt) for miR-29c, a miRNA that targets FZD4, were predicted within *SDNOR* (Fig. 6b). Accordingly, we hypothesized that *SDNOR* induces FZD4 by acting as a sponge for miR-29c. To test this hypothesis, we first investigated the interaction between *SDNOR* and miR-29c. Luciferase assays revealed that miR-29c dramatically decreased the activity of a *SDNOR* reporter vector containing miR-29c binding sites, but had no effect on a control vector containing mutated binding sites (Fig. 6c), confirming that miR-29c binds to its putative binding sites in *SDNOR*. Furthermore, RNA pulldowns confirmed that *SDNOR* physically associates with miR-29c in porcine GCs (Fig. 6d). Together, these data reveal that *SDNOR* acts as a molecular sponge for miR-29c in porcine GCs.

In porcine follicles, the levels of *SDNOR* and miR-29c were negatively correlated (Fig. 6e). Consistent with this, the miR-29c level was significantly elevated in *SDNOR*-KD GCs (Fig. 6f), suggesting that *SDNOR* is a determinant of miR-29c level in porcine ovary cells both in vivo and in vitro. Furthermore, miR-29c decreased both *SDNOR* and FZD4 expression levels in a dose- and time-dependent manner; *SDNOR* was more sensitive than FZD4 to miR-29c (Figs. 6g, h and S9a, b), indicating that *SDNOR* induces FZD4 in porcine GCs by competitively binding miR-29c.

We also investigated whether miR-29c mediates the regulation of downstream molecules and GC apoptosis by *SDNOR*. miR-29c depletion rescued the decrease in FZD4 and β-catenin protein levels caused by si*SDNOR* (Fig. 6i), indicating that miR-29c mediates the regulation of *SDNOR* on FZD4 and the FZD4-dependent Wnt signaling pathway in porcine GCs. We also observed that depletion of miR-29c inhibited GC apoptosis and c-Caspase-3 production induced by *SDNOR* silencing (Fig. 6j), indicating that miR-29c mediates the regulation of GC apoptosis by *SDNOR*. Together, these data demonstrate that *SDNOR* physically associates with miR-29c, thereby alleviating the inhibitory effect of miR-29c on its target FZD4, and subsequently activates the Wnt signaling pathway and inhibits apoptosis in porcine GCs.

## Discussion

In this study, we have clarified the role of SMAD4 in regulating *FZD4* expression in ovarian GCs. We found that SMAD4 acts as a strong inducer of *FZD4*, controlling *FZD4* transcription, the FZD4-dependent Wnt signaling pathway, and FZD4-mediated GC function. In addition, we showed that SMAD4 induces protects FZD4 through two molecular mechanisms: direct interactions with the promoter region of *FZD4* and upregulating a novel lncRNA, *SDNOR*, which acts as a sponge for miR-29c.

The TGF-β signaling pathway is multifunctional, and controls many biological functions related to development and diseases, usually via interactions with other important signaling pathways such as BMP, ERK, Hippo, JAK/STAT, Notch, NF-κB, MAPK, and Wnt signaling pathways^9,47,48^. In particular, crosstalk between the TGF-β and Wnt signaling pathways has been extensively studied^49,50^. However, the regulatory relationship between these pathways in mammalian ovaries, and the specific mechanism involved in their interactions, remain largely unknown^51,52^. In primary human lung fibroblasts, TGF-β1 induces transcription of *FZD7* and *FZD8* in a SMAD3-dependent manner, leading to activation of the Wnt signaling pathway^51,52^. A recent report showed that crosstalk between the TGF-β and Wnt signaling pathways in prostate cancer cells is mediated by an interaction between the cysteine-rich domain of FZD8 and the extracellular domain of TGFBRI^53^. In the present study, we showed for the first time that SMAD4 is a novel strong inducer of *FZD4*, and that both SMAD4 and FZD4 mediate the interaction between the TGF-β and Wnt signaling pathways in porcine GCs. Our findings reveal a novel mechanism of crosstalk between the TGF-β and Wnt signaling pathways, which is mediated by both SMAD4 and FZD4, the core components of these two signaling pathways in mammals.

In mechanistic terms, we discovered two important and unexpected ways that SMAD4 promotes *FZD4* transcription in ovarian GCs. First, SMAD4 directly controls *FZD4* transcription by acting as a transcription factor, the best-characterized manner in which SMAD4 regulates its target genes^12,39,54^. The SMAD4 protein affects the transcriptional activity of target genes, usually by interactions between its DNA-binding domain and SBE motifs within the promoter region of target genes^55^. Intriguingly, the regulatory effect of SMAD4 on target genes can act in one of two opposing ways: it can promote target gene transcription by acting as a transcriptional activator^6,12^, or inhibit the transcription of target genes by acting as a transcriptional repressor^54,56^. In this study, we showed that SMAD4 is a new transcriptional activator of *FZD4*, and that the two factors form a novel regulatory axis that mediates crosstalk between the TGF-β and Wnt signaling pathways in porcine GCs. A recent study has demonstrated that Wnt signaling pathway is the most inactivated pathway in gastric tumors from a Smad4-KO mouse model produced by using a transposon mutagenesis method^36^. Moreover, several components of the Wnt signaling pathway, including Wnt3A52, β-catenin^9,49^, Fzd1, Fzd2^57^, Dkk1, and Sfrp1^58^, as well as downstream targets such as c-Myc and Axin2^58,59^, are regulated by SMAD4. CTNNB1 (also known as β-catenin), for instance, is induced by SMAD4 in human Tenon’s capsule fibroblasts; this factor is involved in the promotion of cell proliferation and activation by TGF-β signaling pathways^9^. Conversely, nuclear β-catenin and other signature genes of the Wnt signaling pathway are upregulated in epithelial tumors harboring Smad4-KO^49^. However, the mechanism by which SMAD4 directly regulates the components of the Wnt signaling pathway had been unclear. Here, we demonstrated that SMAD4 directly binds to the SBE motifs of the FZD4 promoter by acting as a transcription factor, thereby inducing FZD4 and the Wnt signaling pathway in GCs. These findings establish a direct link between the TGF-β and Wnt signaling pathways in GCs.

Second, SMAD4 controls *FZD4* transcription indirectly by promoting expression of *SDNOR*, a novel lncRNA that acts as a competitive endogenous RNA of miR-29c, thereby suppressing the inhibitory efforts of miR-29c on *FZD4* expression at posttranscriptional level. LncRNAs, a class of noncoding transcripts (>200 nt) that lack ORFs, are poorly conserved at the primary sequence level, and are expressed in tissue-specific manners, are implicated in multiple biological processes, and modulate gene expression in *cis* or *trans* with various mechanisms^60,61^. As with miRNAs, many lncRNAs are regulated by the TGF-β signaling pathway^62,63^. A recent study revealed a new mechanism by which TGF-β signaling regulates miRNA biogenesis: *lncRNA nc886*, which is induced by TGF-β, binds to Dicer, a key enzyme involved in miRNA biogenesis, thereby inhibiting miRNA maturation in ovarian cancer^64^. However, the mechanism underlying regulation of lncRNA expression by the TGF-β signaling pathway remains largely unknown. In this study, we identified a novel lncRNA related to follicular atresia, *SDNOR*, and showed that SMAD4 induces *SDNOR* expression in GCs through an interaction with its promoter. In addition to providing a potential nonhormone therapeutic drug for improving follicular atresia and female fertility in pigs, our findings also provide a new insight into the mechanism by which the TGF-β signaling pathway regulates lncRNA expression in mammals. We also showed that SDNOR helps to maintain FZD4 expression by acting as a sponge for endogenous miR-29c, thereby eliminating the inhibition of FZD4 by miR-29c. lncRNA–miRNA interactions are already known to participate in regulation of the Wnt signaling pathway^65^. LncRNA–microRNA systems not only act directly on the core members of the pathway, such as WNT2B^66^, FZD3, and FZD5^65^, but also indirectly on upstream regulators such as GSK3β^67^, SOX9^68^, and APC^69^, thereby affecting the activation of this pathway. Overall, our findings demonstrate that a novel lncRNA–miRNA system, *SDNOR*-miR-29c, mediates positive regulation of FZD4 by SMAD4, establishing a novel molecular link between the TGF-β and Wnt pathways.

**Fig. 7:**
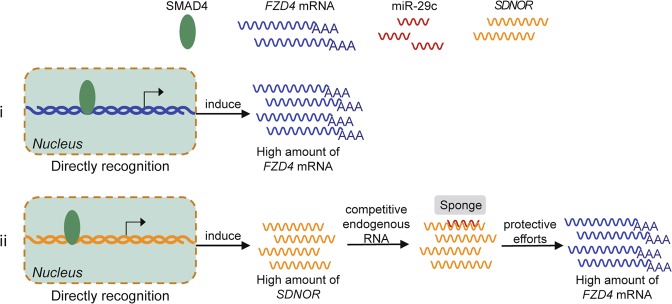
A schematic model for SMAD4 protects FZD4. The schematic diagram depicting the mechanism that SMAD4 protects FZD4 expression and functions: (i) binds to the promoter of *FZD4* and initiates *FZD4* transcription directly; (ii) functions as a transcription factor for a pig novel lncRNA, *SDNOR* which further sponges miR-29c and relieves its suppression to FZD4.

In summary, we have shown that the TGF-β and Wnt signaling pathways engage in crosstalk to regulate GC apoptosis. Specifically, SMAD4 helps to maintain FZD4 expression at both the transcriptional and posttranscriptional level, via both direct and indirect mechanisms, through a complex regulatory network consisting of miRNA, lncRNA, and protein-coding genes (Fig. 7). These coding proteins, especially FZD4, represent promising therapeutic targets for treatment of female reproductive diseases and improvement of female fertility. The noncoding RNAs involved in this pathway, including miR-29c and *SDNOR*, could be developed as nonhormone drugs to regulate these targets.

## Supplementary information

Supplementary Figure and Table Legends

FigureS1

FigureS2

FigureS3

FigureS4

FigureS5

FigureS6

FigureS7

FigureS8

FigureS9

Supplementary Tables
