# Supplementary material for: SMAD4 activates Wnt signaling pathway to inhibit granulosa cell apoptosis
Source: Cell Death Dis. 2020 May 15;11(5):373. doi: 10.1038/s41419-020-2578-x (PMC7228950; doi:10.1038/s41419-020-2578-x)
Supplement: Supplementary file 1 — Supplementary Figure and Table Legends [file 41419_2020_2578_MOESM1_ESM.doc]

**Supplementary Figure Legends**

**Fig. S1.** *FZD4* is differentially expressed during follicular atresia. Detection of *FZD4* mRNA expression levels in pig healthy follicles (HF) and atresic follicles (AF) by qRT-PCR. ^**^*P*<0.01 by two-tailed Student’s *t-*test.

**Fig. S2.** Two SBEs motifs were predicted within *FZD4* promoter. The transcription start site (TSS) of pig *FZD4* was identified using 5’-RACE. The 5’-UTR sequence is depicted in yellow. Transcription factor binding sites are shown with underlines. Two SMAD4 binding elements (SBE1 and SBE2) are depicted in green with black font. ATG is indicated in red font.

**Fig. S3.** Multiple-sequence alignment of miR-29c and *FZD4* 3’-UTR. (a) The multiple-sequence alignment of *pre-miR-29c* from 8 different species. Red asterisks indicate the mature sequence of miR-29c, and the seed sequence of miR-29c are highlighted with green box. (b) Multiple-sequence alignment of *FZD4* 3’-UTR that containing miR-29c response element from six different species. The seed sequence of miR-29c is shown in red.

**Fig. S4.** *FZD4* mRNA level is negatively correlated with miR-29c. *MiR-29c* and *FZD4* expression levels in 12 ovarian follicles were detected and subjected to Pearson correlation analysis. Data were normalized to *GAPDH* expression level and shown as ∆CT (threshold cycle).

**Fig. S5.** miR-29c/FZD4 aixs regulates porcine GC apoptosis. (a) Western blot analysis (left) and quantification (right) of c-Caspase3 protein level in GCs after miR-29c and FZD4 overexpression. (b) Western blot analysis (left) and quantification of c-Caspase3 protein level in GCs after miR-29c and FZD4 knockdown. ^*^*P*<0.05 and ^**^*P*<0.01 by a two-tailed Student’s *t*-test, versus to control or scrambled.

**Fig. S6.** Identification and characterization of pig *SDNOR*. (a) Full length of *SDNOR* transcript was obtained using 5’ and 3’RACE, verified by sequencing. (b, c) The protein-coding probability of *SDNOR* was predicted by CPAT (b) and CPC (c), *GAPDH* and *FZD4* were used as examples of coding genes, *H19* was an example for non-coding gene. (d) Expression levels of *SDNOR* in 4 reproductive tissues (vagina, uterus, oviduct and ovary) from female reproduction system, assessed by qRT-PCR. Data are represented as means ± S.E.M. and at least three separate experiments were conducted.

**Fig. S7.** SMAD4 regulates *SDNOR* expression by affecting its promoter activity. (a) *SDNOR* transcript signals (FPKM value) in GCs after treatment with siRNA (control) or siSMAD4 (SMAD4-KD) according to RNA-seq. (b) *SDNOR* levels in GCs after SMAD4 knockdown, assessed by qRT-PCR. (c) The 5’ regulatory region of *SDNOR*. The candidate promoter of pig *SDNOR* is shown in yellow. SBE motifs are depicted with red font. (d) Schematics depicting recombinant reporter vectors of *SDNOR* promoter region containing wild-type or mutant-type SBEs (top) and their luciferase activities in GCs with or without SMAD4 overexpression. Data are shown as means ± S.E.M. and ^*^*P*<0.05, ^**^*P*<0.01, ns indicates no significance.

**Fig. S8.** SMAD4 regulates the expression and function of FZD4 via *SDNOR*. (a) Western blot analysis (left) and quantification (right) of FZD4 and β-catenin protein level in GCs after FZD4-OE or with *SDNOR*-KD. (b) Western blot analysis (left) and quantification (right) of c-Caspase3 protein level in GCs after FZD4-OE or with *SDNOR*-KD. The center line of box-plot depicts mean. (c) FACS analysis (left) and quantification (right) of apoptosis rate was calculated in GCs treatment with FZD4-OE and *SDNOR*-KD. (d) Western blot analysis (left) and quantification (right) of FZD4 and β-catenin protein level in GCs after SMAD4-KD or with *SDNOR*-OE. (e) Western blot analysis (left) and quantification of c-Caspase3 protein level in GCs after SMAD4-KD or with *SDNOR*-OE. The center line of box-plot depicts mean. (f) FACS analysis (left) and quantification (right) of apoptosis rate was calculated in GCs treatment with FZD4-OE and *SDNOR*-KD. Throughout, data shown as means ± S.E.M.. ^*^*P*<0.05, ^**^*P*<0.01 and ns indicates no significance.

**Fig. S9.** The expression level of miR-29c in porcine GCs. (a) miR-29c expression levels in GCs after treatment with miR-29c mimics at different concentration (0-100 nM), assessed by qRT-PCR. (b) miR-29c expression levels in GCs after transfection with 20 nM miR-29c mimics for different time (0-15 h), assessed by qRT-PCR.

**Supplementary Figure Legends**

**Supplementary Table S1.** Oligonucleotide sequences used in this study

**Supplementary Table S2.** Primers used for vectors construction

**Supplementary Table S3.** Primers used for RACE

**Supplementary Table S4.** Primers used for reverse-transpcription and qRT-PCR

**Supplementary Table S5.** Primers used for ChIP and ChIP-qPCR
