# Supplementary figures and images for: SMAD4 activates Wnt signaling pathway to inhibit granulosa cell apoptosis
Source: Cell Death Dis. 2020 May 15;11(5):373. doi: 10.1038/s41419-020-2578-x (PMC7228950; doi:10.1038/s41419-020-2578-x)

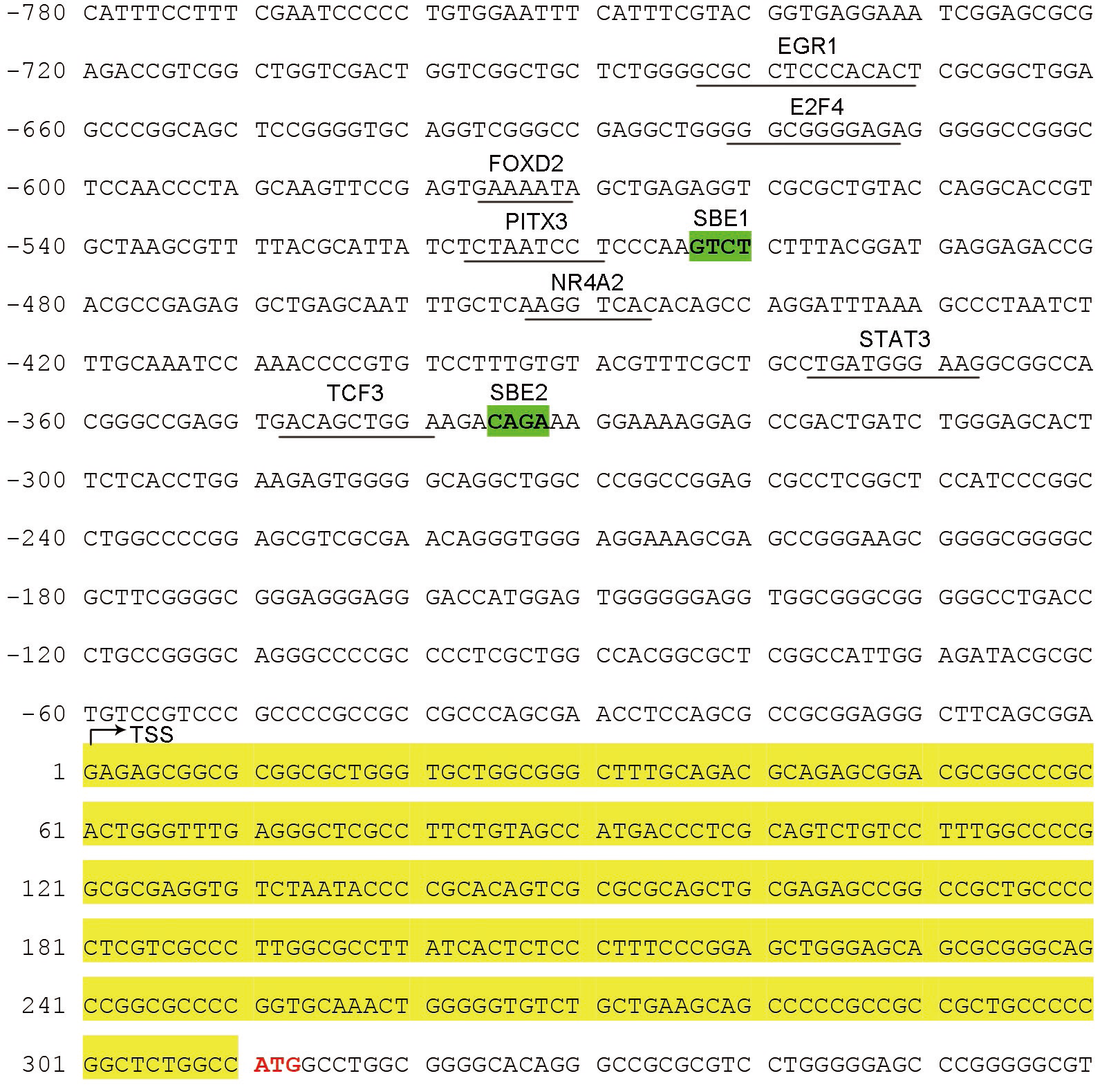

Supplement: Supplementary file 3 — FigureS2 [file 41419_2020_2578_MOESM3_ESM.png]

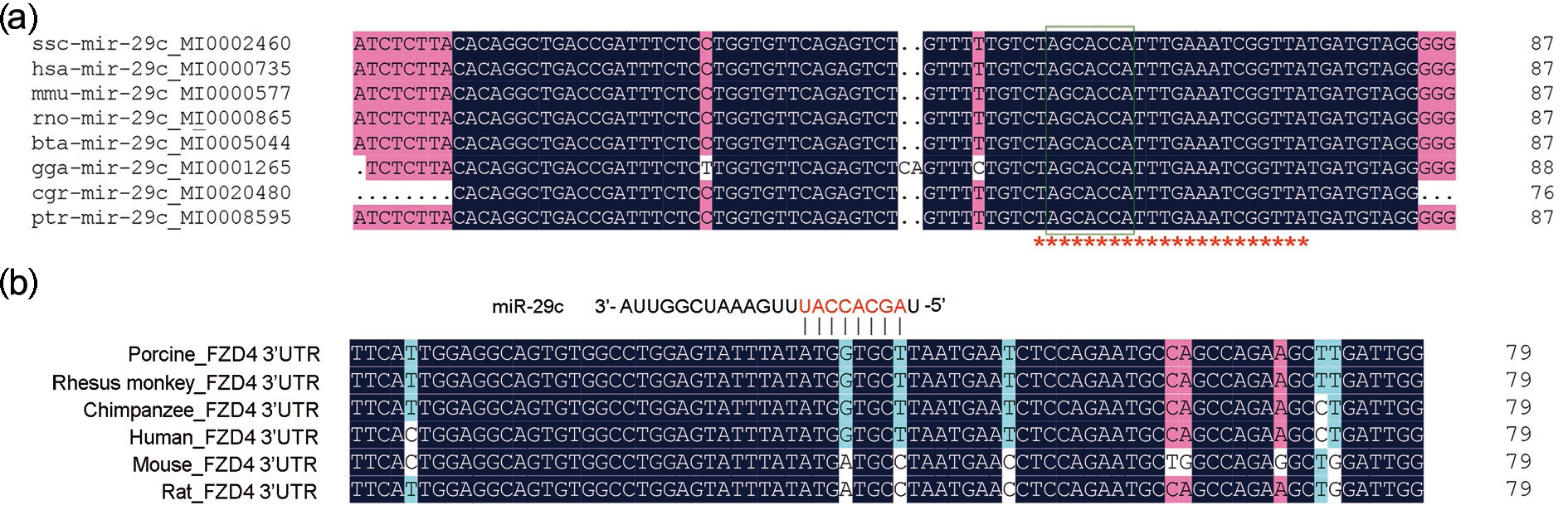

Supplement: Supplementary file 4 — FigureS3 [file 41419_2020_2578_MOESM4_ESM.tif]

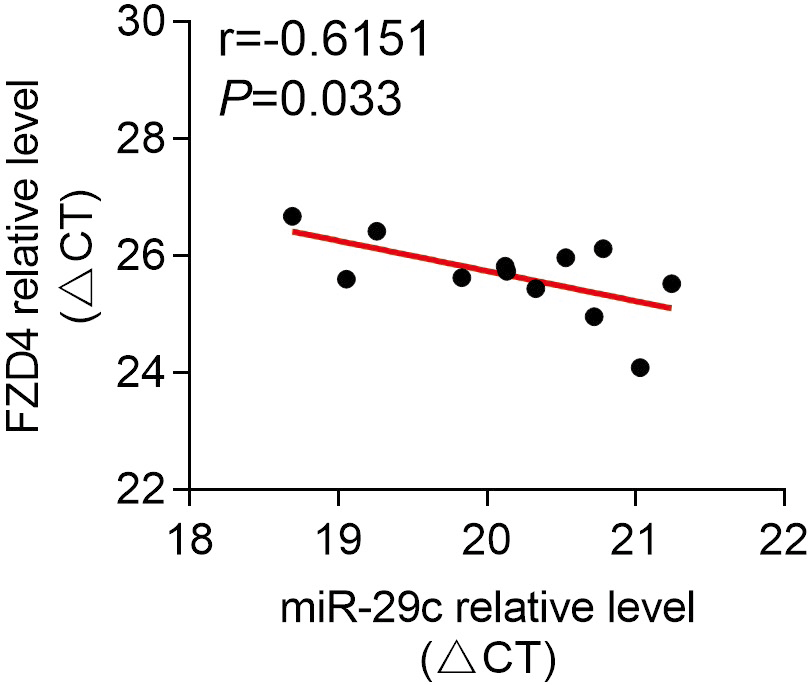

Supplement: Supplementary file 5 — FigureS4 [file 41419_2020_2578_MOESM5_ESM.png]

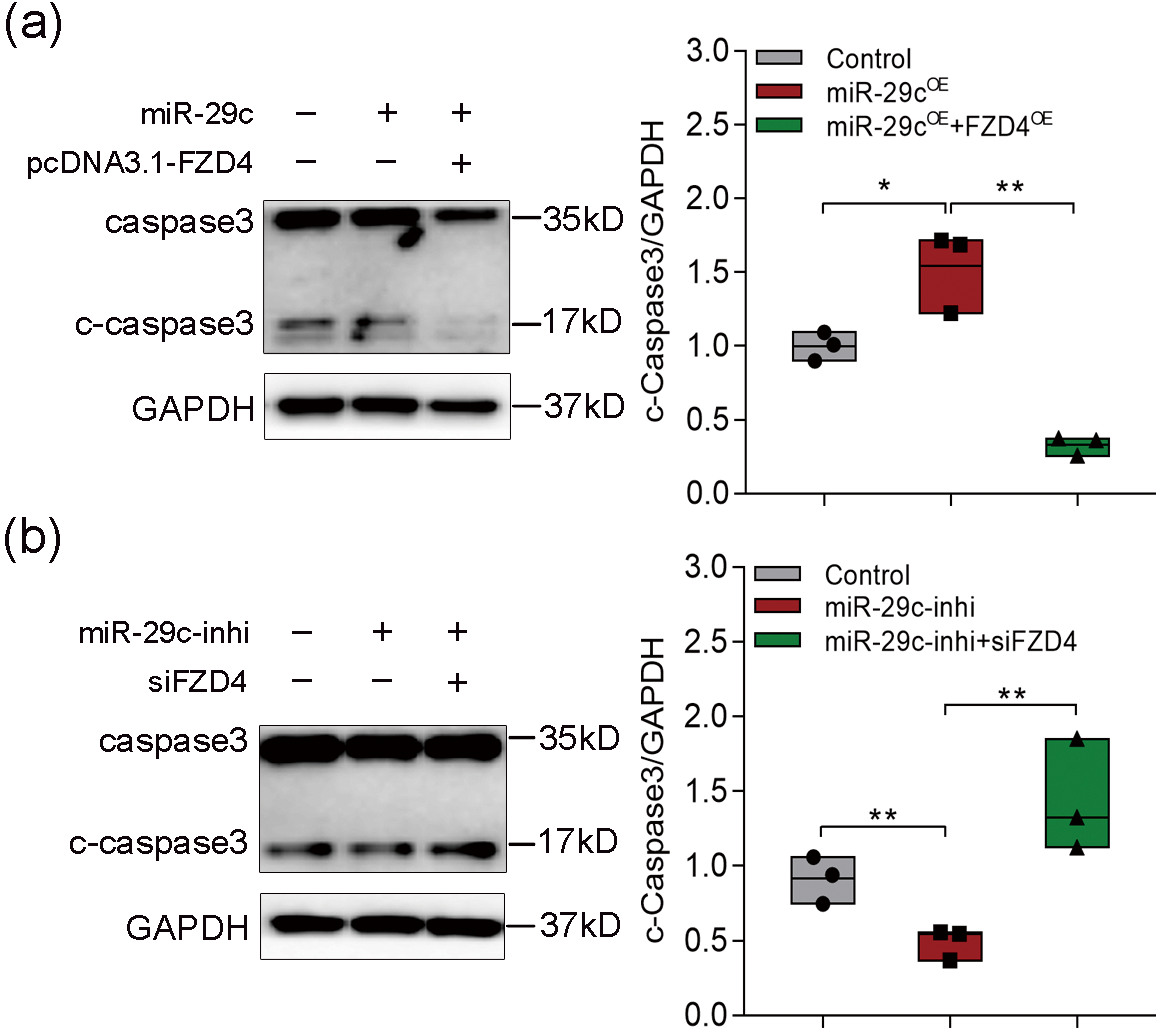

Supplement: Supplementary file 6 — FigureS5 [file 41419_2020_2578_MOESM6_ESM.tif]

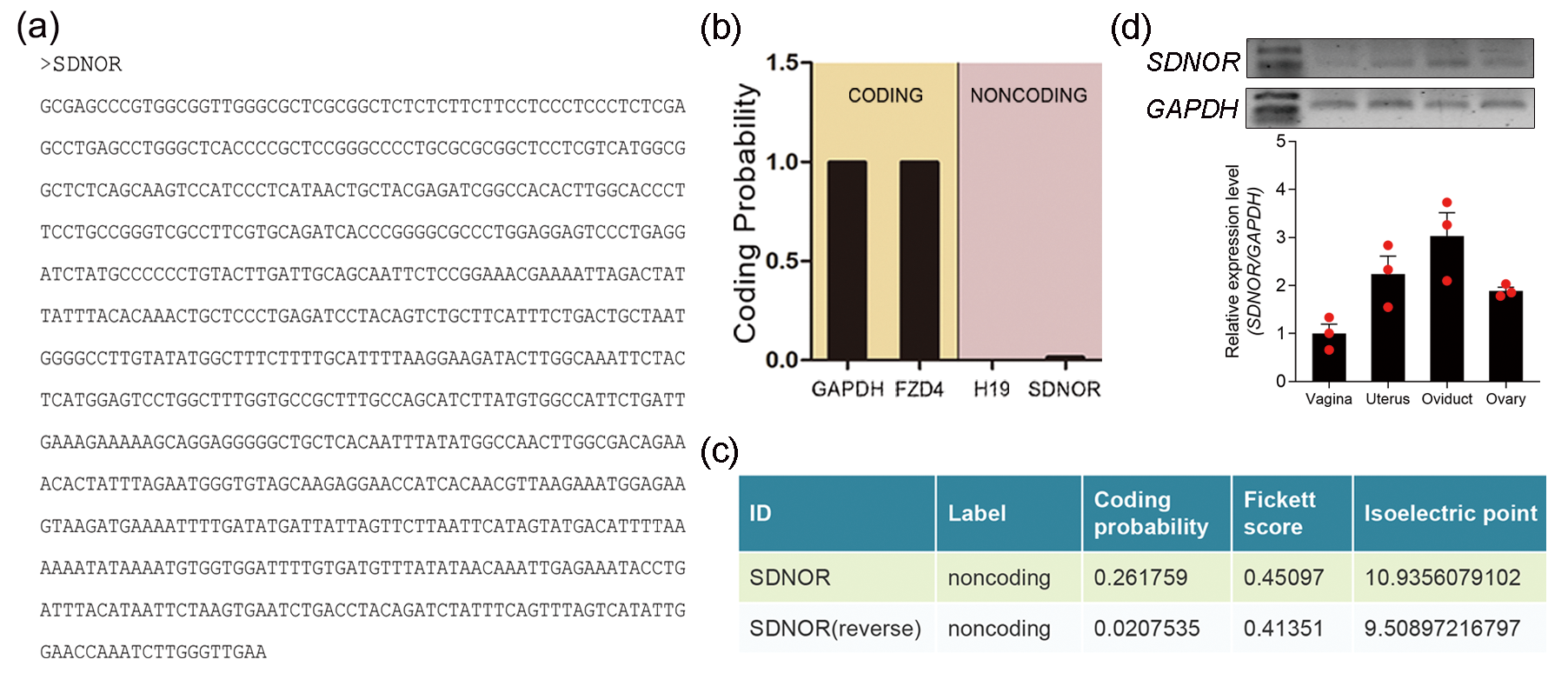

Supplement: Supplementary file 7 — FigureS6 [file 41419_2020_2578_MOESM7_ESM.tif]

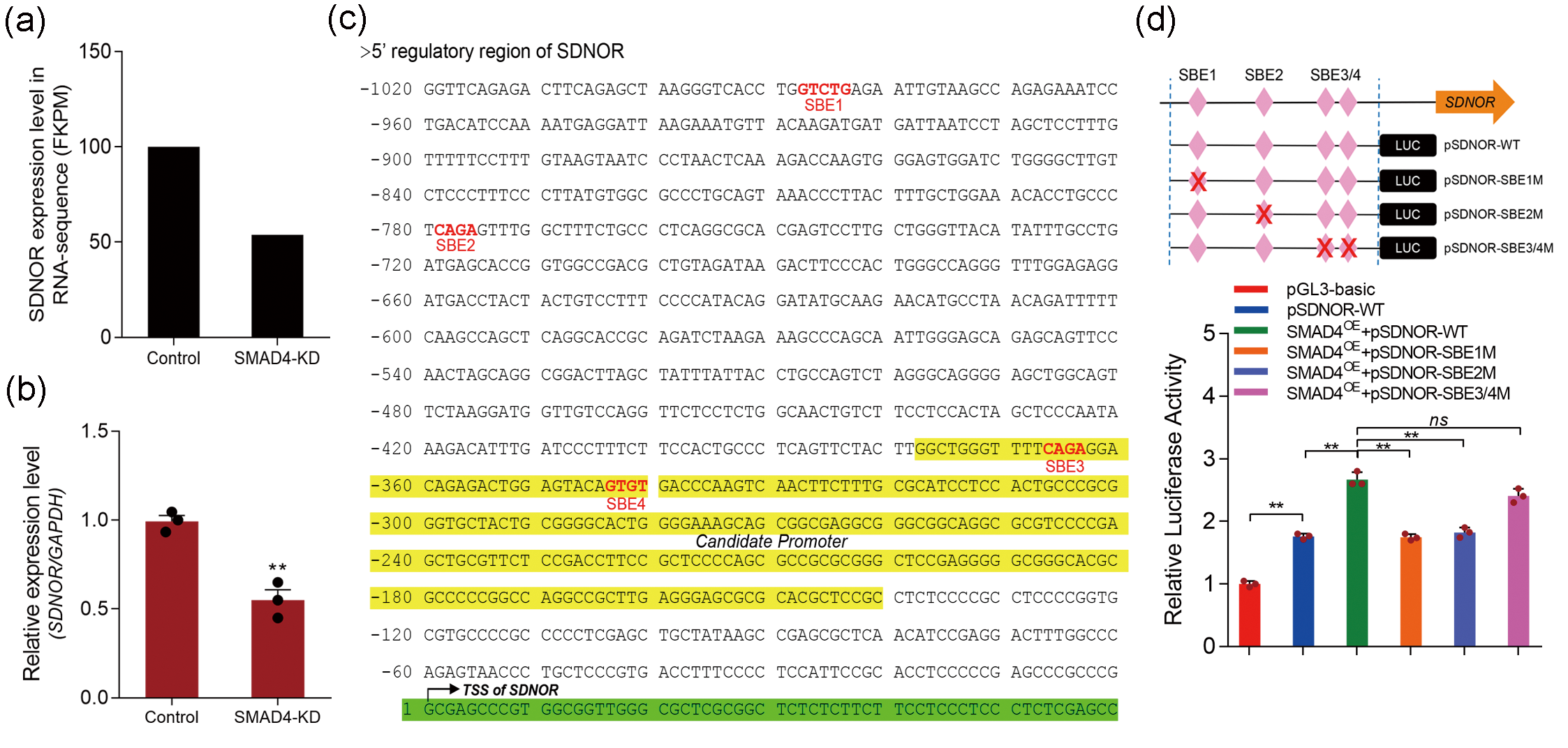

Supplement: Supplementary file 8 — FigureS7 [file 41419_2020_2578_MOESM8_ESM.tif]

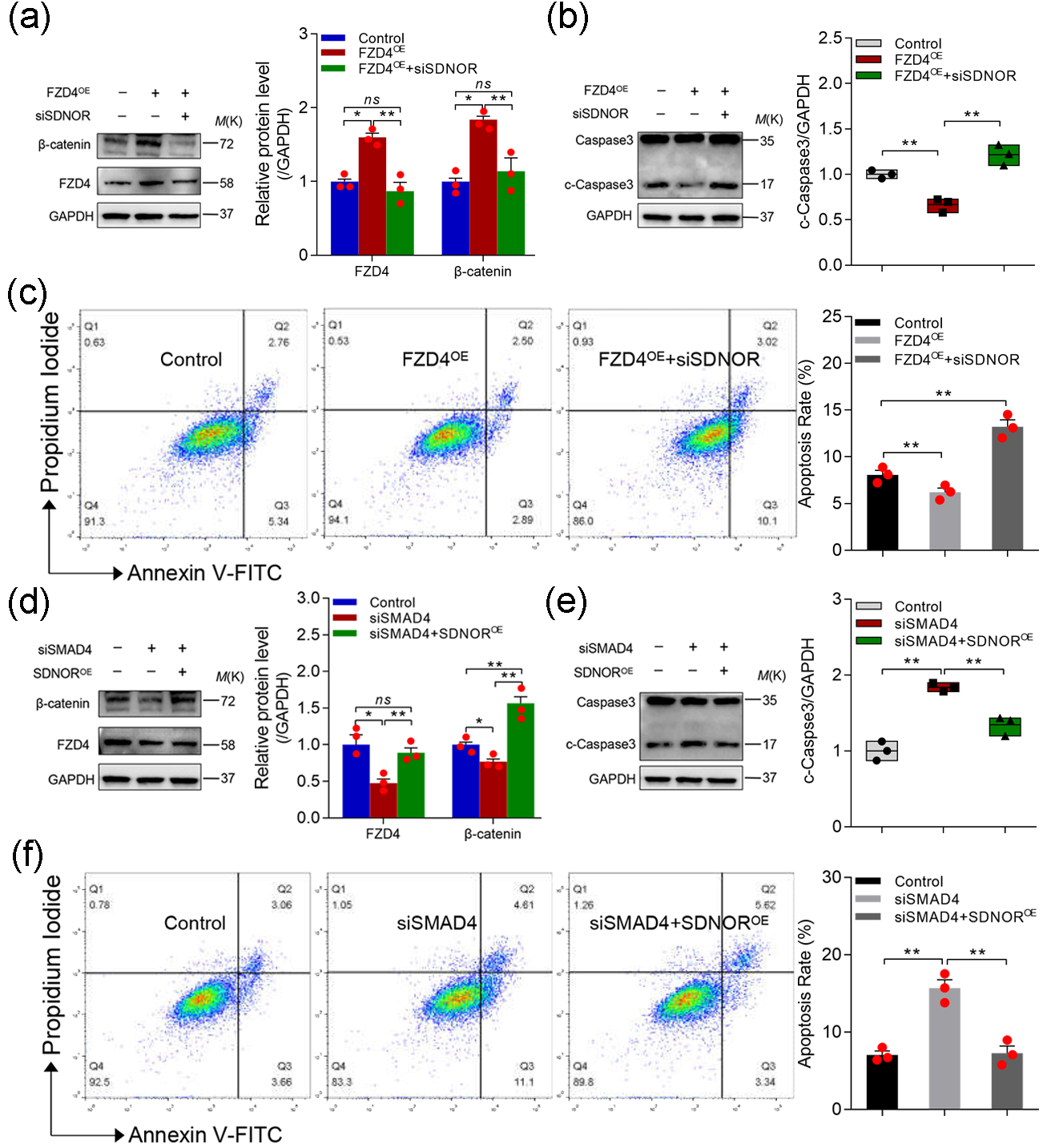

Supplement: Supplementary file 9 — FigureS8 [file 41419_2020_2578_MOESM9_ESM.tif]

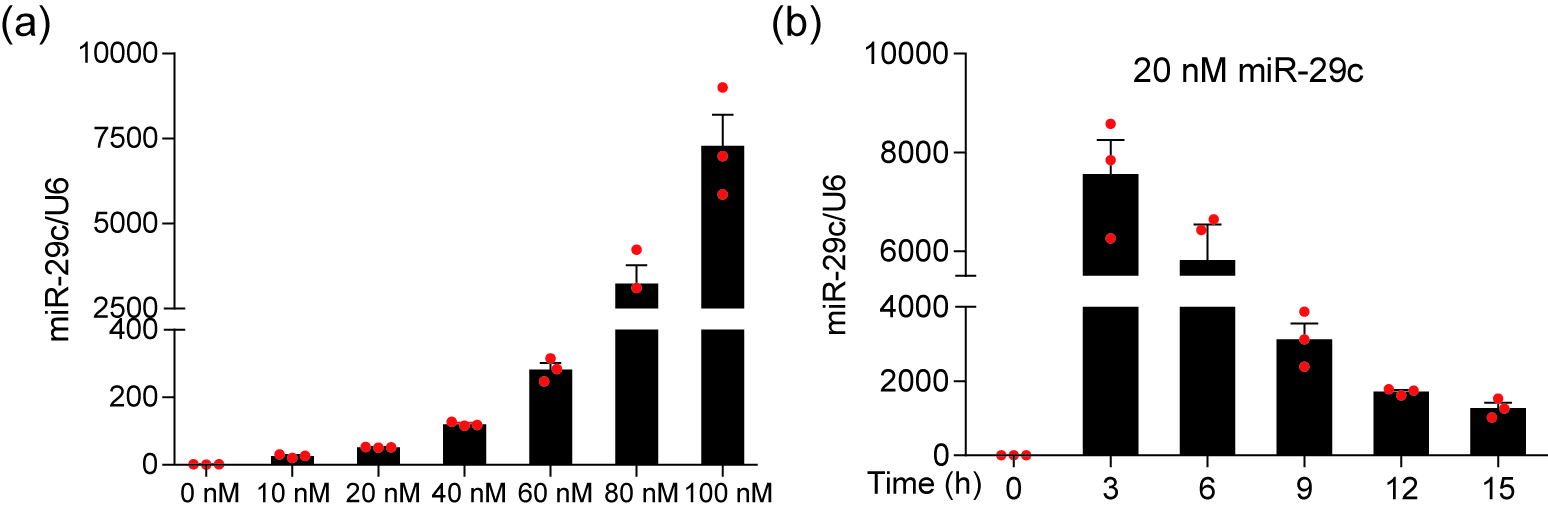

Supplement: Supplementary file 10 — FigureS9 [file 41419_2020_2578_MOESM10_ESM.tif]
