# Supplementary material for: SMAD4 activates Wnt signaling pathway to inhibit granulosa cell apoptosis
Source: Cell Death Dis. 2020 May 15;11(5):373. doi: 10.1038/s41419-020-2578-x (PMC7228950; doi:10.1038/s41419-020-2578-x)
Supplement: Supplementary file 11 — Supplementary Tables [file 41419_2020_2578_MOESM11_ESM.doc]

**Supplementary Tables**

**Supplementary Table S1.** Oligonucleotide sequences used in this study

| **Name** | **Sequences (5’-3’)** | **Source** |
| --- | --- | --- |
| siNC | Sense: UUCUCCGAACGUGUCACGUTT | In this study |
|  | Anti-sense: ACGUGACACGUUCGGAGAATT |  |
| siSMAD4 | Sense:CACCAGGAAUUGAUCUCUCAGGAUU | [36] |
|  | Anti-sense:AAUCCUGAGAGAUCAAUUCCUGGUG |  |
| siFZD4-1 | Sense:GCAUGUGCCUUUCGGUCAATT | In this study |
|  | Anti-sense:UUGACCGAAAGGCACAUGCTT |  |
| siFZD4-2 | Sense:GCCUGAACUGCGUUCUCAATT | In this study |
|  | Anti-sense:UUGAGAACGCAGUUCAGGCTT |  |
| SiFZD4-3 | Sense:GCCAGUUCCAUCUGGUGGGTT | In this study |
|  | Anti-sense:UUGACAACGCAGAUCACGCTT |  |
| si*SDNOR*-1 | Sense:GGAACCAAAUCUUGGGUUGTT | In this study |
|  | Anti-sense:CAACCCAAGAUUUGGUUCCTT |  |
| si*SDNOR*-2 | Sense:GCCACACUUGGCACCCUUCTT | In this study |
|  | Anti-sense:CAACGCUAGUUGCAGAUCCTT |  |
| si*SDNOR*-3 | Sense:GCCAGCAUCUUAUGUGGCCTT | In this study |
|  | Anti-sense:CAACCCAAGAUUUGGUUCCTT |  |
| mimics NC | UUGUACUACACAAAAGUACUG | In this study |
| miR-29c mimics | UAGCACCAUUUGAAAUCGGUUA | In this study |
| Inhibitor NC | CAGUACUUUUGUGUAGUACAA | In this study |
| miR-29c inhibitor | UAACCGAUUUCAAAUGGUGCUA | In this study |

**Supplementary Table S2.** Primers used for vectors construction

| **Plasmids** | **Primers sequence (5’-3’)** | **Vector** | **Usage** |
| --- | --- | --- | --- |
| p*FZD4*-205 | F:TAATACCCCGCACAGTCGC | pGL3-basic | cloning |
| p*FZD4*-424 | F:GCTCGGCCATTGGAGATAC | pGL3-basic | cloning |
| p*FZD4*-629 | F:GTACGTTTCGCTGCCTGATG | pGL3-basic | cloning |
| p*FZD4*-895 | F:CCCTGTGGAATTTCATTTCGTAC | pGL3-basic | cloning |
| p*FZD*4-R | R:GGAGATGCGGATGGGGTC | pGL3-basic | cloning |
| pmir*FZD4*-WT | F:CTAGCTAGCTTTTGTAGAGGGAGCTAAGGA | pmirGLO | cloning |
|  | R:CCGCTCGAGAAGACCTGTGCCGTTAGAGTT | pmirGLO |  |
| pmir*FZD4*-mut | F:CCTGGAGTATTTATACCACCACTAATGAATCTCCAGAATG | pmirGLO | mutation |
|  | R:CCACACTGCCTCCAATGAAATCTTACACCGAGCCTTTGCC | pmirGLO |  |
| p*SDNOR*-SBE1,2 | F:AGACCACTGCTGCCTCTACTATGT | pGL3-basic | cloning |
|  | R:GCAGGTGTTTCCAGCAAAGTAA |  |  |
| p*SDNOR*-SBE3,4 | F:CCTTACTTTGCTGGAAACACCT | pGL3-basic | cloning |
|  | R:GCGGAAGGTCGGAGAACG |  |  |
| p*SDNOR*-SBE1M | F:TAGGCAAGATGGGTTCAGAGACTTCAGAGCTAA | pGL3-basic | mutation |
|  | R:CAGAAGCAAACTGAGCCTGTACGTTAATGATAA |  |  |
| p*SDNOR*-SBE2M | F:GAGAAATCCTGACATCCAAAATGAGGATTAAGA | pGL3-basic | mutation |
|  | R:TGGCTTACAATTCTCCGACCAGGTGACCCTTAG |  |  |
| p*SDNOR*-SBE3M | F:ATTTATTACCTGCCAGCCTAGGGCAGGGGAGCTG | pGL3-basic | mutation |
|  | R:AGCTAAGTCCGCCTGCTAGTTGGAACTGCTCTGC |  |  |
| p*SDNO*R-SBE4M | F:TCTCCTCTGGCAACTGTCGTCCTCCACTAGCTCC | pGL3-basic | mutation |
|  | R:ACCTGGACAACCATCCTTAGAACTGCCAGCTCCC |  |  |
| pmir*SDNOR*-MRE1 | F:GAAGCCCAGGATGTCTCAC | pmirGLO | cloning |
|  | R:GTGGGGATCAGTAAATCAGTTC |  |  |
| pmir*SDNOR*-MRE1M | F:AATTCTACTCATGGAGTCCTGGCTTTGACGCCGCTTT | pmirGLO | mutation |
|  | R:GGCCACATAAGATGCTGGCAAAGCGGCGTCAAAGCCA |  |  |
| pmir*SDNOR*-MRE2 | F:TAACTCCCAGGCGACAGA | pmirGLO | cloning |
|  | R:TCTCCTTCGCTCCACCAT |  |  |
| pmir*SDNOR*-MRE2M | F:ACGTTAAGAAATGGAGAAGTAAGATGAAAATTTTG | pmirGLO | mutation |
|  | R:TGTGATGGTTCCTCTTGCTACTGGCATTCTAAATAG |  |  |

F: forward primers; R: reversed primers.

**Supplementary Table S3.** Primers used for RACE

| **Name** | **Primers sequence (5’-3’)** |
| --- | --- |
| *FZD4* 5’RACE | GATTACGCCAAGCTTAGCACGGTGAAGGCGGTGGAGATGAA |
| *SDNOR* 5’RACE | GATTACGCCAAGCTTTGGGTGTAGCAAGAGGAACCATCACAAC |
| *SDNOR* 3’RACE | GATTACGCCAAGCTTAATAGTGTTTCTGTCGCCAAGTTGGCC |

**Supplementary Table S4.** Primers used for reverse-transpcription and qRT-PCR

| **Gene** | **Primers sequences (5’-3’)** | **Usage** |
| --- | --- | --- |
| *FZD4* | F:ACCTGGGCTACAACGTGAC | qRT-PCR |
|  | R:ACACCGAGCAAAGGAAGAA |  |
| *SDNOR* | F:CAACTTGGCGACAGAAACACT | qRT-PCR |
|  | R:TTTGGTTCCAATATGACTAAACTGA |  |
| *GAPDH* | F:GGACTCATGACCACGGTCCAT | qRT-PCR |
|  | R:TCAGATCCACAACCGACACGT |  |
| *RPLP0* | F:TCCAGGCTTAGGCATCACC | qRT-PCR |
|  | R:GGCTCCCACTTTGTCTCCAG |  |
| *U6* | F:GCTTCGGCAGCACATATACT | qRT-PCR |
|  | R:TTCACGAATTTGCGTGTCAT |  |
| *miR-29c* | F:CGGGCUAGCACCAUUUGAAA | qRT-PCR |
|  | R:CAGCCACAAAAGAGCACAAT |  |
| *Pre-miR-29c* | F:ATCTCTTACACAGGCTGACCGA | qRT-PCR |
|  | R:TCCCCCTACATCATAACCGATT |  |
| *miR-126* | F:CGGGCCACCACCTCCCCTGCAA | qRT-PCR |
|  | R:CAGCCACAAAAGAGCACAAT |  |
| *miR-130a-3p* | F:CGGGCCAGUGCAAUGUUAAA | qRT-PCR |
|  | R:CAGCCACAAAAGAGCACAAT |  |
| *miR-29c* | CCTGTTGTCTCCAGCCACAAAAGAGCACAATATTTCAGGAGACAACAGGTAACCGA | Reverse-transcrpition |
| *miR-126* | CCTGTTGTCTCCAGCCACAAAAGAGCACAATATTTCAGGAGACAACAGGCGCATTA | Reverse-transcrpition |
| *miR-130a-3p* | CCTGTTGTCTCCAGCCACAAAAGAGCACAATATTTCAGGAGACAACAGGATGCCCT | Reverse-transcrpition |

**Supplementary Table S5.** Primers used for ChIP and ChIP-qPCR

| **Name** | **Primers sequences (5’-3’)** |
| --- | --- |
| *FZD4*-SBE1 | F:CAAGTTCCGAGTGAAAATAGC |
|  | R:CTGGCTGTGTGACCTTGAG |
| *FZD4*-SBE2 | F:GTGTACGTTTCGCTGCCTGAT |
|  | R:CAGGTGAGAAGTGCTCCCAGAT |
| *SDNOR*-SBE1 | F:CTTCTGACCTCAGTCCTGTG |
|  | R:CTGAACCCATCTTGCCTAC |
| *SDNOR*-SBE2 | F:CTTCTGTAGGCAAGATGGG |
|  | R:TCCACTCCCACTTGGTCTT |
| *SDNOR*-SBE3/4 | F:ATCTAAGAAAGCCCAGCAA |
|  | R:GAGGAAGACAGTTGCCAGA |
| SBEX | F:CAGCAAAGGGTGGCAAGGT |
|  | R:ATGCCTGTGCCACTTTCAACT |
